# Transforming Growth Factor Beta (TGF-β) Is a Muscle Biomarker of Disease Progression in ALS and Correlates with Smad Expression

**DOI:** 10.1371/journal.pone.0138425

**Published:** 2015-09-16

**Authors:** Ying Si, Soojin Kim, Xiangqin Cui, Lei Zheng, Shin J. Oh, Tina Anderson, Mohammad AlSharabati, Mohamed Kazamel, Laura Volpicelli-Daley, Marcas M. Bamman, Shaohua Yu, Peter H. King

**Affiliations:** 1 Department of Neurology, University of Alabama at Birmingham, Birmingham, Alabama, United States of America; 2 Birmingham VA Medical Center, Birmingham, Alabama, United States of America; 3 Department of Biostatistics, University of Alabama at Birmingham, Birmingham, Alabama, United States of America; 4 Department of Cell, Developmental and Integrative Biology, University of Alabama at Birmingham, Birmingham, Alabama, United States of America; 5 Department of Genetics, University of Alabama at Birmingham, Birmingham, Alabama, United States of America; Boston University School of Medicine, UNITED STATES

## Abstract

We recently identified Smads1, 5 and 8 as muscle biomarkers in human ALS. In the ALS mouse, these markers are elevated and track disease progression. Smads are signal transducers and become activated upon receptor engagement of ligands from the TGF-β superfamily. Here, we sought to characterize ligands linked to activation of Smads in ALS muscle and their role as biomarkers of disease progression. RNA sequencing data of ALS muscle samples were mined for TGF-β superfamily ligands. Candidate targets were validated by qRT-PCR in a large cohort of human ALS muscle biopsy samples and in the G93A SOD1 mouse. Protein expression was evaluated by Western blot, ELISA and immunohistochemistry. C2C12 muscle cells were used to assess Smad activation and induction. TGF-β1, 2 and 3 mRNAs were increased in ALS muscle samples compared to controls and correlated with muscle strength and Smads1, 2, 5 and 8. In the G93A SOD1 mouse, the temporal pattern of TGF-β expression paralleled the Smads and increased with disease progression. TGF-β1 immunoreactivity was detected in mononuclear cells surrounding muscle fibers in ALS samples. In muscle cells, TGF-β ligands were capable of activating Smads. In conclusion, TGF-β1, 2 and 3 are novel biomarkers of ALS in skeletal muscle. Their correlation with weakness in human ALS and their progressive increase with advancing disease in the ALS mouse suggest that they, as with the Smads, can track disease progression. These ligands are capable of upregulating and activating Smads and thus may contribute to the Smad signaling pathway in ALS muscle.

## Introduction

A hallmark of motor neuron degeneration in amyotrophic lateral sclerosis (ALS) is progressive muscle weakness and atrophy. In the ALS mouse, pathological changes can first be detected in muscle and the neuromuscular junction prior to motor neuron loss, indicating that these structures can reflect early disease activity [1–3]. We recently identified members of the Smad family (Smads1, 5, and 8) as muscle biomarkers of disease progression in ALS [4]. These Smads are upregulated and activated in the ALS mouse at an early stage, before overt clinical signs are present, suggesting they may also be markers of disease onset. Smads are signal transducers for members of the transforming growth factor beta (TGF-β) superfamily which includes TGF-β and bone morphogenic proteins (BMP) [5–7]. Ligands in this family bind to type I and II receptors at the cell surface to form an active complex which leads to the phosphorylation (activation) of specific receptor-regulated Smads. Activated Smads complex with Smad4 and translocate to the nucleus and modulate gene transcription or miRNA processing [6, 8]. In this report, we show significant increases in TGF-β1, 2, and 3 mRNAs in human and mouse ALS muscle tissue that correlate with the Smads, including Smad2. These molecular changes were observed in the pre-clinical stages of the G93A SOD1 mouse in parallel with the Smads. For TGF-β1, the source appeared to be mononuclear cells surrounding muscle fibers. In cultured muscle cells, these ligands were capable of inducing and activating the Smads. These findings characterize a potential axis of Smad activation in ALS muscle tissue, and suggest that TGF-β isoforms, as with Smad1, 2, 5, and 8, are markers of disease progression and possibly disease onset.

## Materials and Methods

### Animals

B6.Cg-Tg (SOD1*G93A) 1 Gur/J mice were purchased from The Jackson Laboratory (Bar Harbor, ME). Transgenic mice were maintained in the hemizygous state by mating G93A males with C57BL/6J females. Non-transgenic littermates were used as controls. For the G93A SOD1 mice, clinical progression was evaluated by weight determination and performance on the rotarod as described previously [4]. End stage disease was determined when the mouse could not right itself after 30 seconds when placed on its side. At this point animals were euthanized by CO2 inhalation followed by cervical dislocation. All animal procedures were reviewed and approved by the UAB Institutional Animal Care and Use Committee in compliance with the National Research Council Guide for the Care and Use of Laboratory Animals.

### Tissue Collection and Cell Culture

The study was approved by the UAB Institutional Review Board. Muscle biopsy samples were identified in a database of archived samples from the neuromuscular division at UAB. The clinical details of patients with ALS, neuropathy, myopathy or no neuromuscular disease are described in our previous publication [4]. C2C12 cells were grown in high glucose DMEM containing 10% FBS, 1% 100, and 1 mM sodium pyruvate. After splitting cells, the medium was changed to high glucose DMEM containing 2% horse serum, and 1 mM sodium pyruvate. Cells were treated with TGF-β1, 2 and 3 (R&D System) at 10 ng/ml.

### Western Blot, ELISA and Immunohistochemistry

For Western blot, tissues were homogenized in T-Per (Pierce Endogen, Rockford, IL) and quantitated with a bicinchoninic acid (BCA) protein assay kit. Sixty micrograms of protein were electrophoresed in an SDS-polyacrylamide gel, blotted and probed with antibodies to the following targets: TGF-β1 (Promega, Madison, WI), TGF-β2 (Santa Cruz Biotechnology, Paso Robles, CA), TGF-β3 (Abcam, Cambridge, MA), and GAPDH (Cell Signaling, Danvers, MA). Densitometry was done with the VersaDoc Imaging System (Bio-Rad, Hercules, CA) and quantified using Image Lab (Bio-Rad). For immunohistochemistry, ten micron paraffin sections and OCT sections were used. OCT slides were fixed in Bouin’s fixative for 15 min. Deparaffinized sections were immersed in 10 mM citrate buffer (pH 6.0) heated at 100°C for 30 min, and allowed to cool to room temperature. After fixation, all sections were treated with 3% H_2_O_2_ for 10min. After blocking, sections were incubated with TGF-β1, 2 and 3 antibodies (1:50) overnight at 4°C. Slides were incubated for an hour at RT with donkey anti-rabbit secondary antibody conjugated with horseradish peroxidase-labeled polymer (Jackson ImmunoResearch, West Grove, PA). Slides were incubated in TSA Plus Cyanine 3 (PerkinElmer, Waltham, MA) at 1:1,500 for 30 min, Wheat Germ Agglutinin (WGA), Alexa Fluor® 488 (Invitrogen, Carlsbad, CA) at 1:400 for 20 min, and Hoechst 33342 (Sigma-Aldrich, St. Louis, MO) at 1:20,000 for 5 min. The prepared slides were viewed under a TCS SP5 Visible-Upright Confocal Microscope (Leica Microsystems, Buffalo Grove, IL). TGF-β1 quantities in mouse and human muscle lysates were determined using the human TGF-β1 Quantikine ELISA Kit (R&D System) according to the manufacturer's instructions. Samples were acid activated using a protocol provided by the manufacturer. Quantities were estimated based on a standard curve generated with recombinant TGF-β1.

### RNA Isolation and qRT-PCR

RNA was extracted from frozen tissues with Trizol Reagent (Invitrogen) according to the manufacturer’s instructions. Two micrograms of RNA were reverse transcribed according to the manufacturer’s specifications (Applied Biosystems). Multiplex PCR was done using as previously described [4].

### Statistical Analyses

Comparisons between ALS group and controls were conducted using a Mann Whitney test. A Student’s t test was used for analysis of mouse qRT-PCR and ELISA results. For Western blot analysis, we first normalized the densitometry values with the housekeeping control (GAPDH) for each lane. A paired t test was used for assessing densitometry comparisons within each blot, comparing wild-type to G93A samples at each age. Pearson correlation coefficients were calculated using Graphpad software (San Diego, CA).

## Results

### TGF-β1, 2 and 3 mRNAs Are Elevated in Muscle Biopsies of Patients with ALS

**Fig 1:**
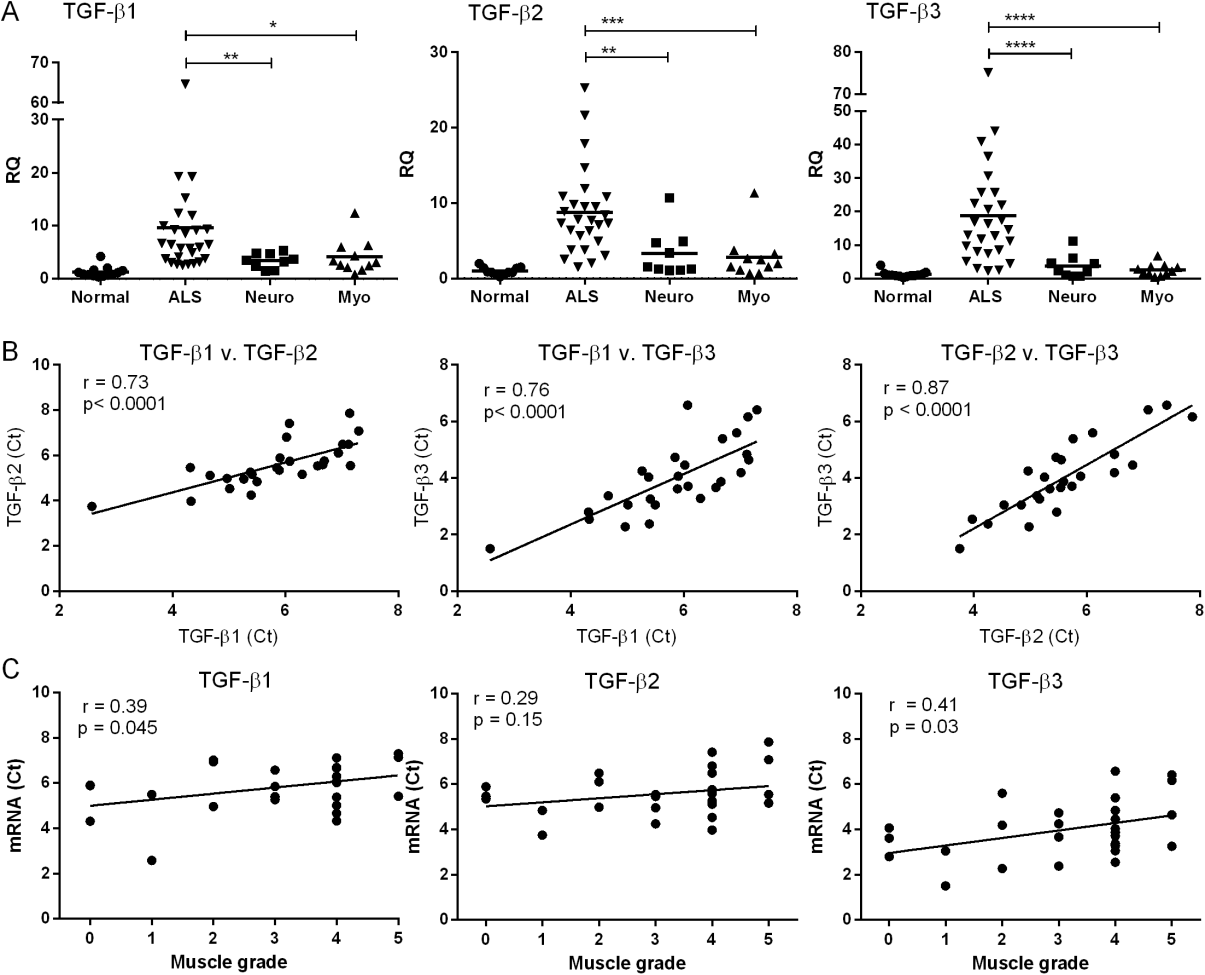
TGF-β mRNA is increased in muscle samples from ALS patients. (A) Total RNA from muscle biopsy samples was analyzed by qRT-PCR for TGF-β1, 2, and 3 mRNA expression in patients with ALS (n = 27), myopathy (n = 11), neuropathy (n = 9) or no neuromuscular disease (n = 13). RQ, relative quantity. *, p < 0.05; **, < 0.005; *** < 0.0005; **** < 0.0001. (B) Correlation of TGF-β isoform mRNA levels (expressed as the Ct value from qRT-PCR). (C) Correlation between muscle grade of biopsied ALS muscle samples (as measured by the Medical Research Council scale) and TGF-β mRNA.

**Fig 2:**
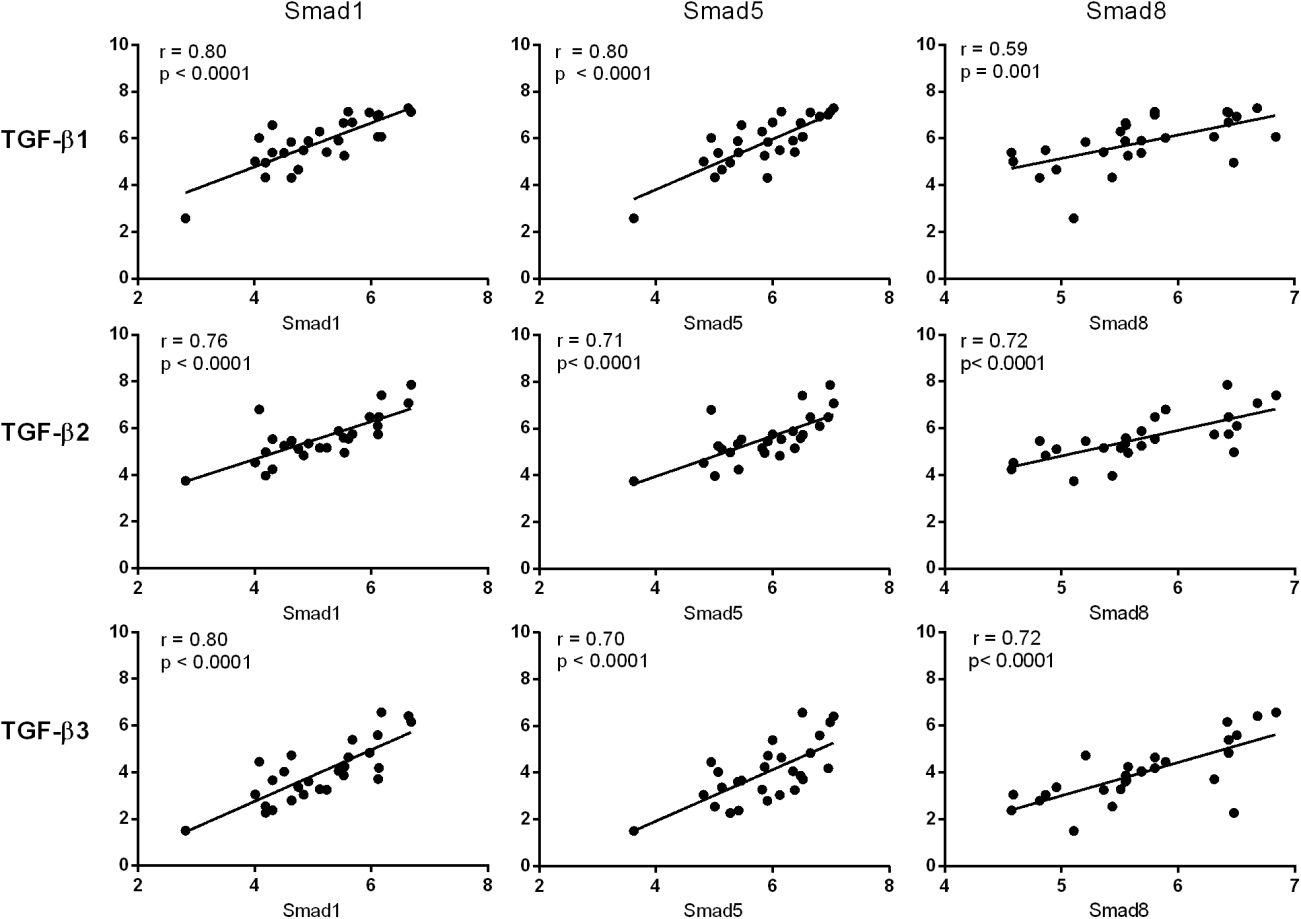
TGF-β and Smad mRNA levels correlate in ALS muscle samples. Smad1, 5 and 8 mRNA levels were determined by qRT-PCR [4] and compared with TGF-β mRNA levels from the same ALS muscle biopsy sample (Ct values are shown).

In our previous report, we observed phosphorylation (activation) and upregulation of Smads1, 5 and 8 in ALS muscle tissue [4]. These signal transduction proteins are typically activated after engagement of a TGF-β ligand to its cognate cell surface receptor. An analysis of our RNA sequencing data (methodology reported previously [4]) revealed a significant increase in TGF-β3 mRNA in ALS muscle biopsy samples. We assessed this target in a cohort of 27 ALS patients and compared expression levels to controls (Fig 1A). Clinical descriptions of the cohort and controls were previously described [4]. Briefly, the ALS group had a mean age of 61 years, equally divided between males and females, with ~25% bulbar and 75% spinal onset. Myopathy controls consisted of patients with polymyositis and mitochondrial myopathy; neuropathy controls included patients with axonal and demyelinating peripheral neuropathies. We observed a 15-fold increase over normal controls and a ~5 to 7-fold increase over neuropathy and myopathy controls (p < 0.0001). TGF-β1 and 2 mRNAs, although not identified by RNA sequencing, also increased (2 to 3-fold) over diseased controls. There was significant correlation among the different isoforms in the ALS samples (p < 0.0001; Fig 1B). An inverse correlation was observed between muscle grade (Medical Research Council scale) of the biopsied muscle and TGF-β1 and 3 mRNA levels (Fig 1C). We next compared Smad mRNA levels to TGF-β and found striking positive correlations between Smads 1, 5 and 8 with each isoform (Fig 2). Pearson coefficient values were 0.70 or higher for all but one of the comparisons (p < 0.0001). In summary, the RNA data show that TGF-β1, 2 and 3 mRNAs are significantly increased in muscle of ALS patients, and this expression pattern parallels that of the Smads.

### TGF-β mRNA and Protein Are Increased at an Early Age in the G93A SOD1 Mouse

**Fig 3:**
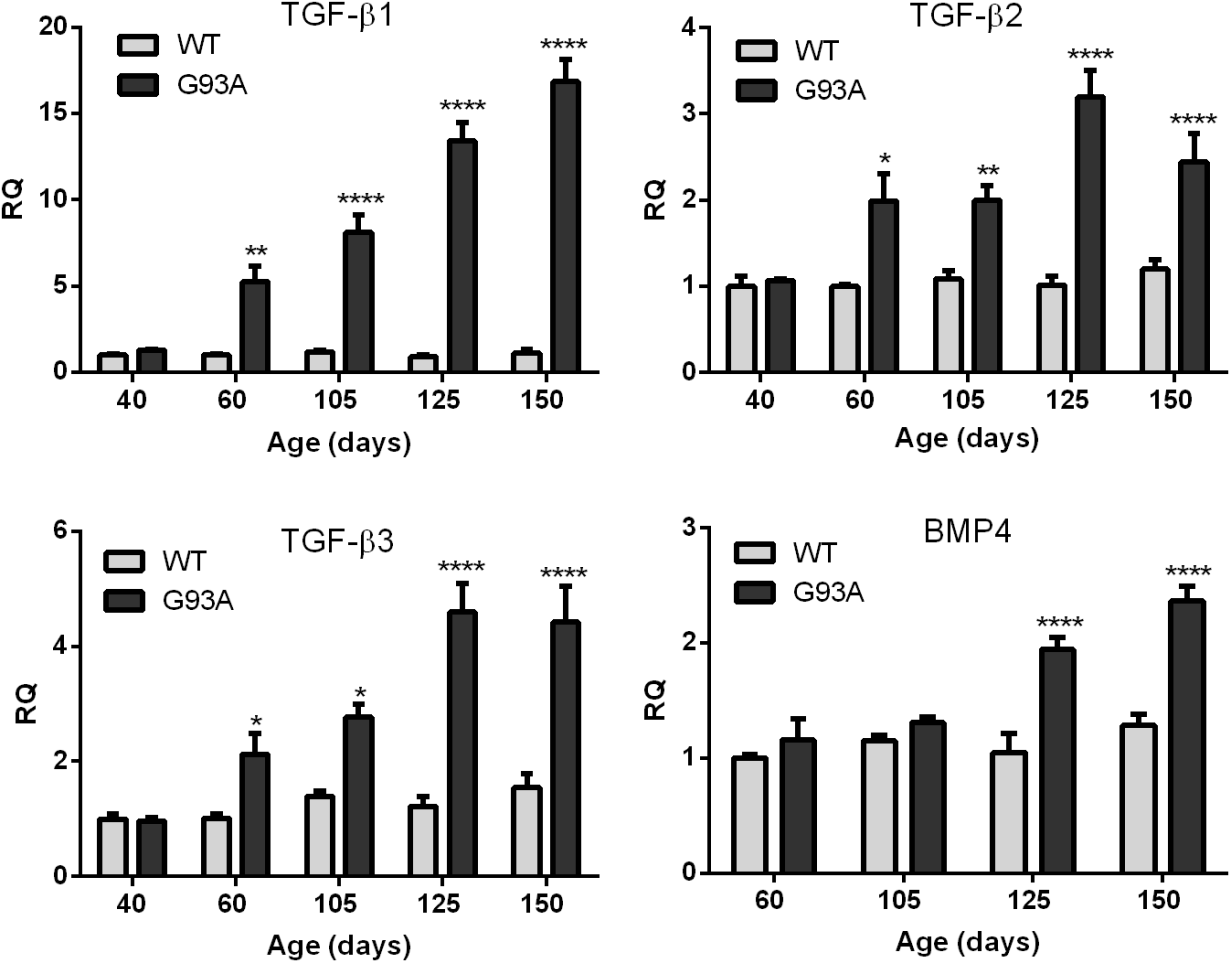
TGF-β mRNA levels are increased at early stages of ALS in the G93A SOD1 mouse. Total RNA was isolated from G93A SOD1 mice and littermate controls (WT) at 40, 60 and 105 d (preclinical stages as previously defined [4]), and early and late clinical stages (125 and 150 d). Samples were analyzed by qRT-PCR for TGF-β1, 2, 3 and BMP4 mRNA expression. Data points represent the mean ± SE of 6–8 mice. * p < 0.05; ** < 0.005; **** < 0.0001. RQ, relative quantity.

**Fig 4:**
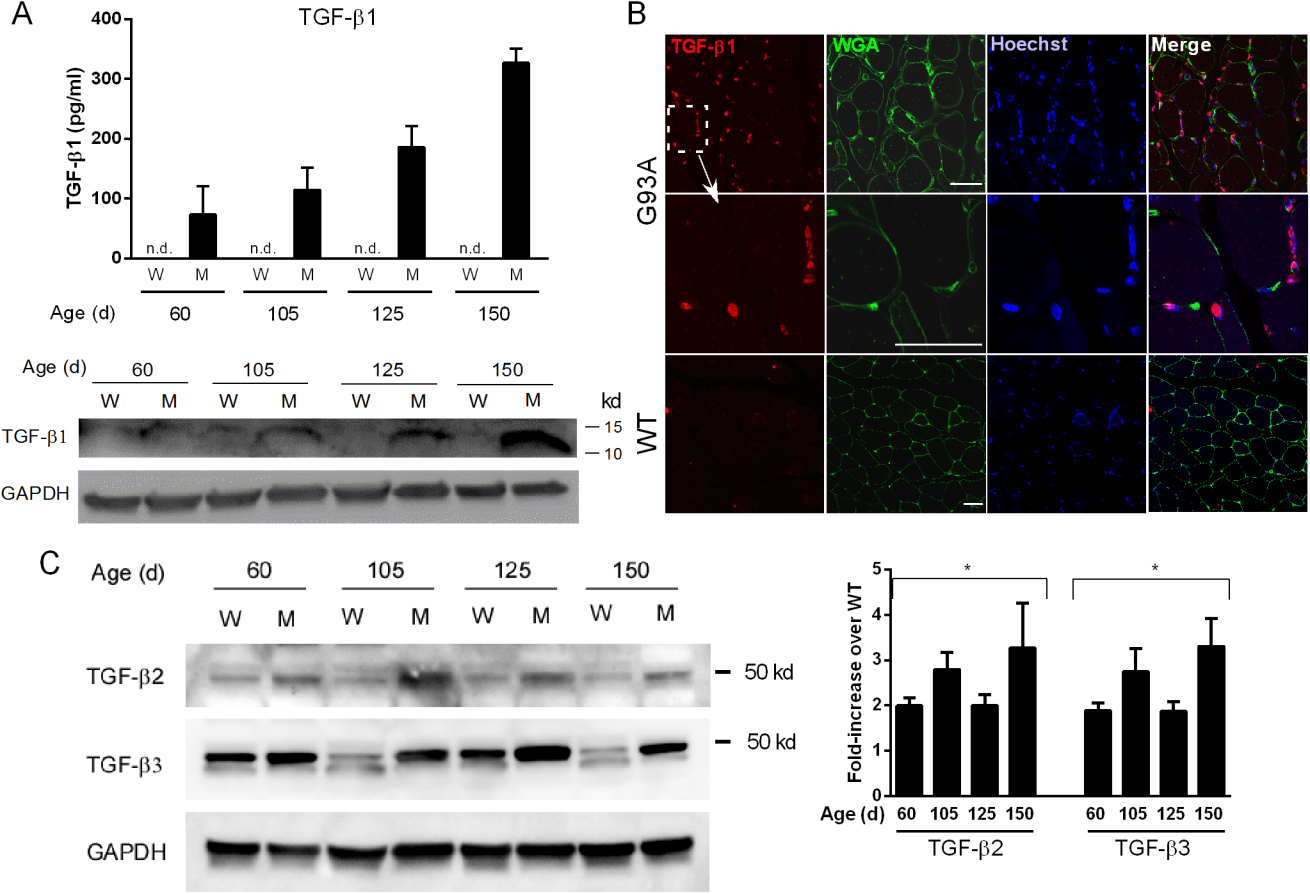
TGF-β protein is elevated in G93A mouse muscle. (A) Muscle lysates from G93A SOD1 mice (M) and non-transgenic littermates (W) were assessed for TGF-β1 by ELISA and Western blot. ELISA values were determined by comparison to a standard curve. A representative Western blot of the lysates (under reducing conditions) shows expression of mature TGF-β1. ELISA data represent the mean ± SE of 3 mice. The Western blot was repeated once with the similar results. (B) Confocal photomicrographs of G93A or WT muscle sections using a TGF-β1 antibody. WGA, wheat germ agglutinin. Size marker = 50 microns. (C) A representative Western blot of TGF-β2 and β3 in mouse muscle showing increased levels of the unprocessed peptide in mutant versus control samples. Quantitative densitometry of TGF-β ligands was performed on three Western blots from three independent mouse samples. Data are shown as fold-increase over WT controls. *p < 0.05 for each age.

We next determined whether TGF-β mRNA upregulation occurred in skeletal muscle from the G93A SOD1 mouse and if the temporal pattern of expression paralleled that of the Smads. In our previous report, we observed induction of Smad1, 5 and 8 mRNAs between post-natal day 40 and 60 [4]. At that time interval, no overt clinical manifestations are observed, but subtle motor deficits have been described [9]. In our colony, the animals have a longer survival time (mean of 161 days) and no gender effect (data not shown). These features are similar to what has previously been reported for this model [9]. Further details regarding progression and disease onset are provided elsewhere [4]. For all three TGF-β isoforms we observed a significant increase in mRNA compared to littermate controls starting at 60 d and throughout the clinical course (Fig 3). TGF-β1 showed the greatest increase at each time point (more than 15-fold by end-stage). There was no increase at 40 d in any of the isoforms indicating a temporal pattern similar to the Smads [4]. BMP4, another TGF-β receptor ligand which showed a non-significant upward trend in our RNA sequencing analysis, increased only in the later stages (125 and 150 d) indicating that TGF-β1, 2 and 3 mRNA induction was relatively selective in the early stages of disease. We next assessed protein expression. For TGF-β1, we observed gradual increases by ELISA with disease progression (Fig 4A). Detection required acid activation indicating that the ligand is predominantly in the latent form [10]. Western blot (under reducing conditions) showed a similar increase in the mature form of TGF-β1 with disease progression. Immunohistochemistry with a TGF-β1 antibody showed labeling of mononuclear cells adjacent to muscle borders as outlined by WGA staining (Fig 4B). Little to no staining was observed in WT muscle. For TGF-β2 and 3, Western blot analysis showed an increase of pre-processed protein in mutant mice over control at each age toward end-stage. Densitometry of three independent mouse samples indicated a 2–3-fold increase over age-matched controls, with an upward trend at end-stage (Fig 4C). Processed forms were not detected, and immunohistochemistry did not show consistent staining for either isoform (not shown).

### TGF-β1 Protein Is Expressed in Human ALS Muscle

**Fig 5:**
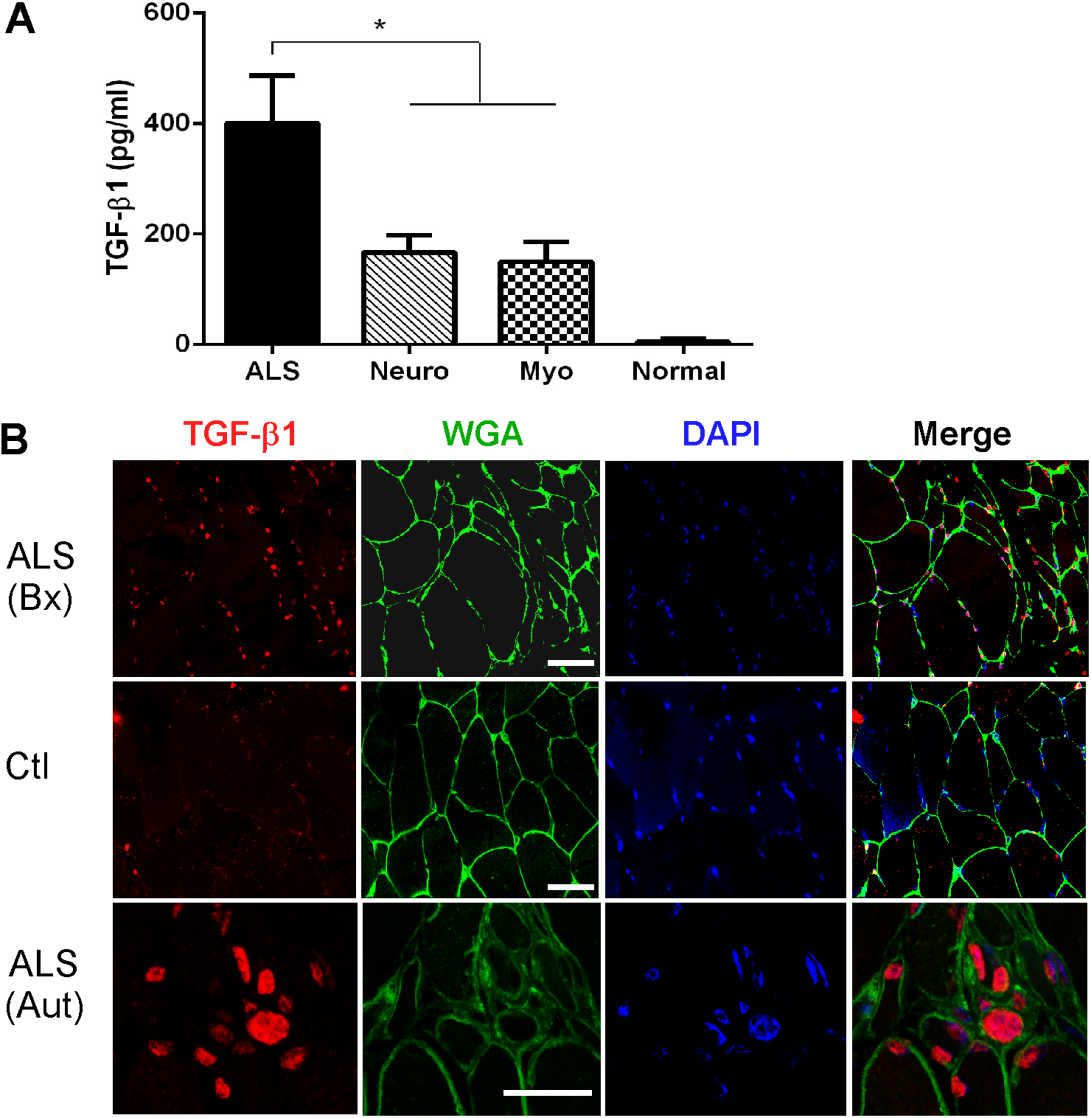
TGF-β1 is increased in human ALS muscle. **(**A) Acid activated protein lysates from human muscle biopsy samples were assessed for TGF-β1 by ELISA and compared to a standard curve. Data points are the mean ± SE. Samples include ALS (n = 12); neuropathy (n = 7); myopathy (n = 8); normal (n = 4). *, p < 0.05. (B) Confocal photomicrographs of ALS and control muscle samples labeled with an anti-TGF-β1 antibody. Bx, biopsy specimen; Aut, autopsy specimen; Ctl, normal biopsy. Size marker = 50 microns.

ELISA analysis of muscle lysates showed a marked increase in TGF-β1 in human ALS samples that was greater than 2-fold over disease controls (Fig 5A). Little to no protein was detected in normal muscle biopsy samples. As with the mouse samples, acid activation of the lysates was required to detect expression. For TGF-β2 and 3, we were not able to detect protein expression by ELISA (not shown). To determine the location of TGF-β1, we performed immunofluorescence on ALS and control muscle biopsy specimens (Fig 5B, upper two rows). We observed a pattern similar to mouse ALS muscle, with immunoreactivity identified in numerous mononuclear cells adjacent to myofiber borders, and only scant staining in control sections. A second human muscle sample from a patient with end-stage ALS revealed a cluster of labeled cells in an area of grouped atrophic fibers (lowest row).

### TGF-βs Induce Smads1,5 and 8 in C2C12 Muscle Cells

**Fig 6:**
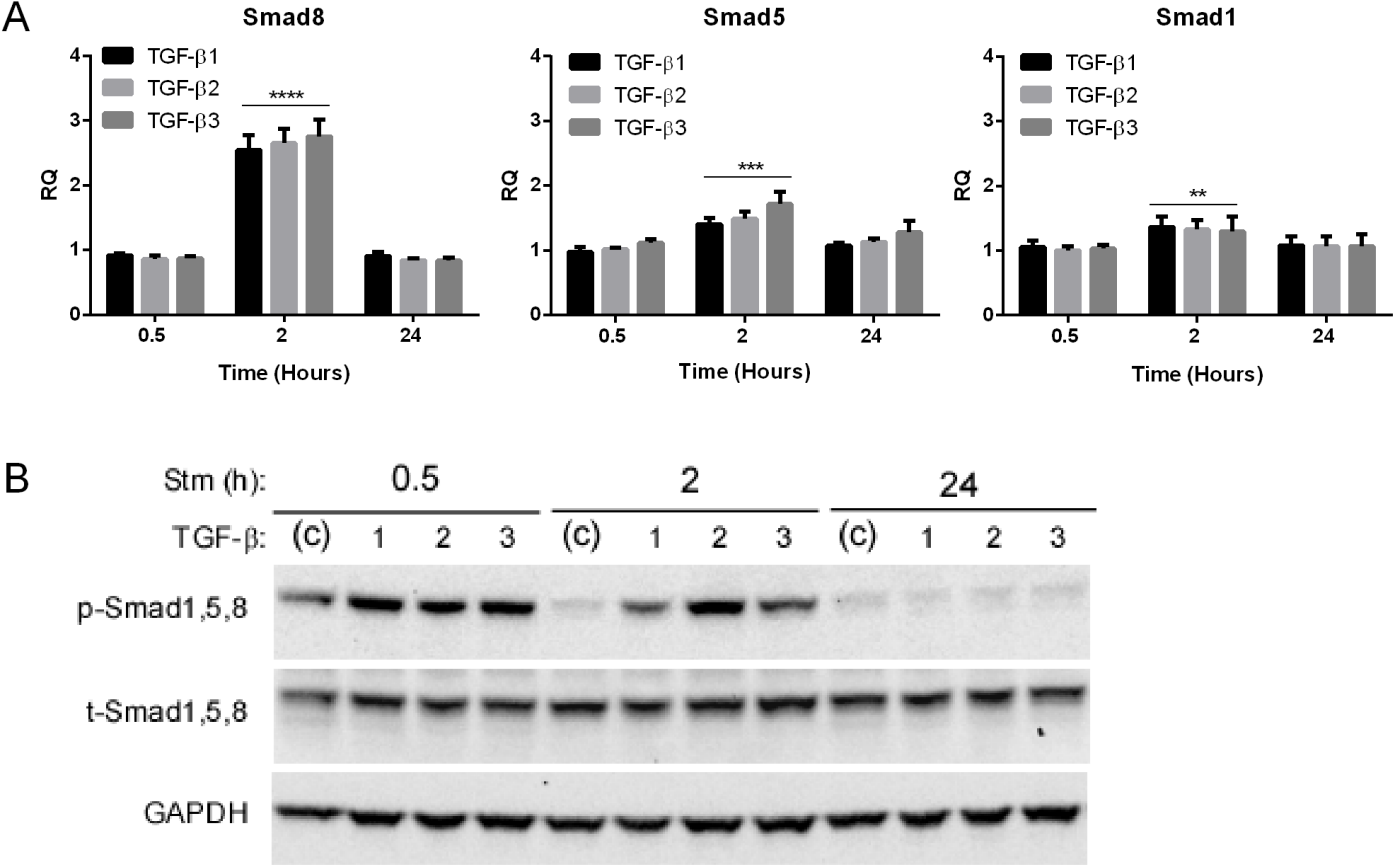
TGF-β induces Smads1, 5 and 8 in cultured C2C12 muscle cells. (A) C2C12 muscle cells were treated with TGF-β ligands for the time frames shown and then assessed for Smad1, 5, and 8 mRNA expression by qRT-PCR. Data points were expressed as a fold-increase over vehicle treated cells and represent the mean ± SE of 6–8 independent samples. ** P < 0.005; *** < 0.0005; **** < 0.0001. (B) C2C12 cells were treated with TGF-β ligands for the time frames shown and assessed for p- and t-Smad 1, 5, 8 by Western blot. The experiment was repeated one time with similar results.

To determine a potential link with Smad1,5 and 8 induction and activation, we stimulated C2C12 muscle cells with TGF-β ligands at different time intervals and assessed Smad mRNA and protein (Fig 6). We observed a transient and significant induction of mRNA with all three isoforms, most prominently with Smad8, at two hours post stimulation. Smad8 showed a nearly 3-fold increase versus a more modest 1.3 to 1.5-fold increase with the Smad1 and 5. Interestingly, this differential pattern of expression is similar to what we observed in mouse and human ALS muscle samples where Smad8 was significantly higher than the other two mRNAs [4]. We next looked at phosphorylated (p)-Smad1, 5, 8 which is the activated form (Fig 6B). By 0.5 h there was marked induction of p-Smad1, 5, 8 equally by all three TGF-β isoforms which persisted through 2 h but dissipated by 24 h. Total Smad1, 5, 8 did not change with TGF-β stimulation in contrast to the increase we observed in the ALS mouse tissue [4]. In summary, TGF-β stimulation of muscle cells in culture recapitulated some of the patterns observed with the Smads in human and mouse ALS muscle.

### Smad2 Is Increased in ALS Muscle

**Fig 7:**
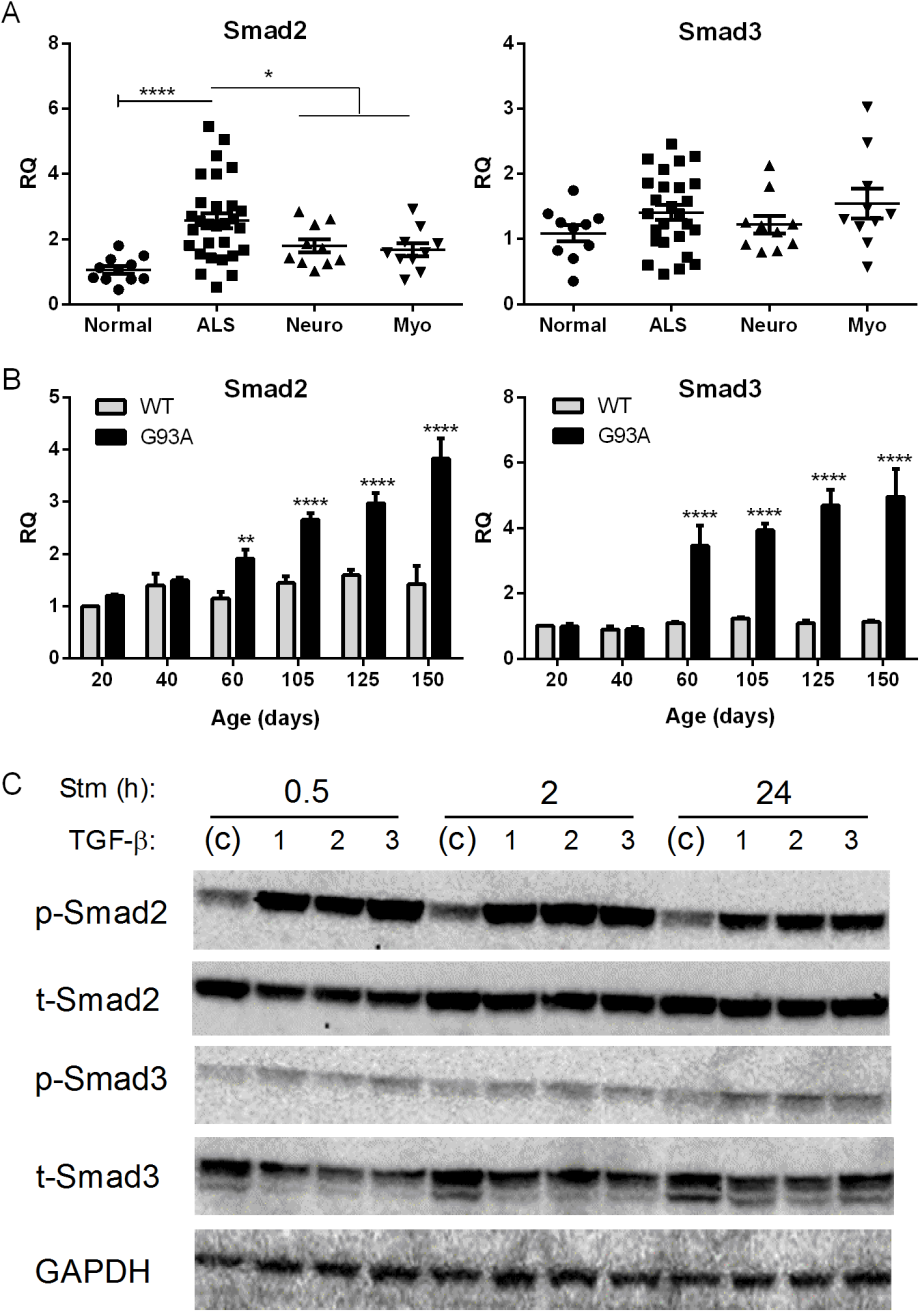
Smad2 and 3 are increased in ALS muscle. (A) Total RNA from human muscle biopsy samples was analyzed by qRT-PCR for Smad2 and 3 mRNA expression as described in Fig 1. (B) Smad2 and3 mRNA levels in the G93A mouse were determined by qRT-PCR. Data points represent the mean ± SE of 3–4 mice. (C) Western blot of C2C12 cells treated with TGF-β ligands for the times shown. Antibodies are shown to the left. RQ, relative quantity. *, P < 0.05; ** < 0.005; ****< 0.0001.

Although our original RNA sequencing analysis did not show significant elevation of Smads2 and 3 over disease controls, they are more typically linked with TGF-β for activation. We assessed our muscle biopsy samples by qRT-PCR and observed a significant, albeit a much smaller fold-increase (< 2-fold), in Smad2 mRNA in ALS versus disease controls (Fig 7A). Smad3, on the other hand, was not elevated compared to disease or normal control specimens. In the G93A mouse both targets were elevated beginning at 60 d post-natal and progressively increased toward end-stage (Fig 7B). In C2C12 muscle cells, all three TGF-β isoforms robustly induced p-Smad2 at 0.5 and 2 h which persisted at 24 h (Fig 7C). Smad3 on the other hand showed a more modest activation at the different time intervals. Neither Smad2 nor Smad3 mRNA was induced with TGF-β stimulation (not shown).

## Discussion

Here we have shown that TGF-β1, 2 and 3 isoforms are significantly increased at the mRNA and protein levels in human and mouse ALS muscle samples. The early and progressive increase of TGF-β, particularly isoforms 1 and 3, in the ALS mouse indicates that these targets are biomarkers of disease progression and potentially disease onset. Interestingly, TGF-β1 and 3 mRNA levels correlated with the degree of weakness of the biopsied muscle, suggesting that these targets may reflect the severity of disease involvement in human ALS. The striking correlation between Smads1, 5, and 8 and TGF-β in human samples (Fig 1), coupled with a similar temporal expression in the ALS mouse (Fig 3 and Ref. 4), raises the possibility of a biological link. Smads1, 5, and 8 are typically phosphorylated by activin receptor-like kinase (ALK) 1, 2, 3 and 6 receptors, after ligand engagement and complexing with type II receptors, whereas ALK 4, 5 and 7 phosphorylate Smads2 and 3 [6, 7]. Although BMP ligands classically activate the ALK receptors linked to Smad1, 5, and 8 phosphorylation, there is clear evidence of crosstalk with TGF-β ligands [6, 11–14]. In our report, we showed that all three TGF-β isoforms could activate Smads1, 5 and 8 in cultured muscle cells and induce the mRNA (Fig 6). The induction of Smad8 mRNA, moreover, exceeded that of Smads1 and 5 by two-fold in a pattern similar to what we previously observed in human and mouse ALS muscle samples [4]. Interestingly, BMP4 regulates Smad9 (the ortholog of Smad8) expression in Xenopus during embryonic development. Analysis of BMP4 in the large cohort of ALS patients in this study failed to show any significant differences (not shown). Furthermore, in the ALS mouse we observed an increase in BMP4 mRNA only in the late stages of disease, well after Smad induction/activation takes place (Fig 3). Smad 2 which is a classic transducer of TGF-β1, 2 and 3 signaling was not identified in our original RNA sequencing likely because of the lower fold-increase over disease controls (< 2-fold). Its increase in human muscle samples, the strong correlation with TGF-β mRNA levels, and the progressive increase in the ALS mouse starting at a time point similar to the other Smads (60 d) indicate that this family member could also be a biomarker of disease progression. Smad3, however, was only increased in the animal model, so its relevance to human disease is less clear. As expected, TGF-β stimulation of muscle cells led to activation of both Smad2 and 3. In light of the many other potential TGF-β ligands, however, it remains to be seen whether TGF-β1, 2 or 3 in mouse or human ALS muscle drive Smad activation [6, 7].

While our data showed the presence of mature TGF-β1, it was in the latent form based on the requirement of acid activation for detection by ELISA. For TGF-β2 and 3, we only saw the precursor form on Western blot in mouse tissues but were not able to detect expression by ELISA in mouse or human tissues. Processing of TGF-β ligands is a complex, multi-step process [10, 15, 16]. Mature TGF-β is produced by proteolytic cleavage of the latency-associated peptide (LAP) located at the N-terminal. After cleavage, LAP complexes with mature TGF-β to mute its activity by blocking sites that interact with TGF-β receptors [10, 15, 16]. This complex typically resides in the extracellular matrix, and activation of mature TGF-β occurs when factors such as plasmin, integrins or thrombospondin disrupt the association with LAP. Detection of active TGF-β ligands in muscle tissues may well be below the sensitivity of the assays used here [17]. Outside of skeletal muscle, there have been reports of increased TGF-β1 in plasma and CSF of patients with ALS, and a positive correlation with disease duration [18]. Others have shown increases of TGF-β1 mRNA in spinal cord tissues from the G93A SOD1 mouse [19, 20]. The elevation of TGF-β1 protein (and mRNA) is not specific for ALS as we observed a significant increase of this isoform in muscle samples of neuropathy and myopathy patients (Figs 1 and 5). Thus, the utility of these markers, as with the Smads, lies in their capacity to reflect progression of disease more so than specific diagnosis. However, their early appearance, prior to overt clinical manifestations, may be useful for staging the disease.

TGF-β1 was detected in mononuclear cells surrounding muscle fibers (Figs 5 and 6), suggesting that Smads in ALS muscle fibers may be activated through a paracrine effect. Previous reports describe TGF-β expression in multiple compartments of muscle including the terminal branches of the motor neuron, Schwann cells, muscle fibers and neuromuscular junction [21]. In a model of nerve injury, TGF-β2 was upregulated at the mRNA level and found to be increased at the protein level in extrasynaptic regions of muscle fibers [22]. In a small group of ALS patients some immunoreactivity was detected in atrophic fibers but considerably less than primary muscle disease [23]. Although the nature of the cells in our report is currently being investigated, a central question regards the potential role TGF-β ligands are playing in ALS muscle. Smad signaling in muscle plays divergent roles depending on the pathological context and the pathway activated. In denervation states, such as with nerve injury, activation of Smads1,5 and 8 is essential for hypertrophic signaling [24, 25] whereas Smad2/3 activation promotes muscle atrophy [26]. Some reports show that TGF-β1 and 2 can promote motor neuron survival [27–29]. Motor neurons express type I and II TGF-β receptors in their axons and nerve terminals which may transduce both autocrine and paracrine TGF-β signaling [21, 30]. Systemic administration of TGF-β2 produced some therapeutic benefit in the SOD1 mouse although it did not prolong survival [31]. Thus the presence of TGF-β in the milieu may be beneficial or harmful in ALS. In primary muscle disorders such as muscle injury, muscular dystrophy or Marfan syndrome, TGF-β signaling impedes muscle regeneration [32, 33]. This effect may stem from an inhibition of satellite cell proliferation and myofiber fusion [32]. TGF-β1 also promotes fibrosis in these disorders by inducing differentiation of myogenic cells to fibroblasts [32, 34–36]. Although increased connective tissue and/or fibrosis has been observed in ALS muscle histopathologically, [37, 38] this was not a prominent feature in our biopsy specimens.

## Conclusions

TGF-β1, 2, and 3 are significantly increased in human and mouse ALS muscle and parallel the Smads. The correlation with muscle strength in the human specimens coupled with an increase in expression with clinical advancement in the ALS mouse suggests that these ligands are markers of disease progression. They may also have utility in diagnosis or clinical staging as they appear well before overt clinical weakness in the ALS mouse. Although multiple muscle biopsies to test for these markers in ALS patients would not be practical, it is possible that these ligands could be detected in blood or imaged by a target-specific contrast agent or radiopharmaceutical. The biological role of an activated Smad signaling pathway in ALS muscle is unclear and will require further investigation to determine whether it is beneficial or harmful.
